# Outcomes of rechargeable sacral neuromodulation for faecal incontinence: A single‐centre observational study

**DOI:** 10.1111/codi.70344

**Published:** 2025-12-21

**Authors:** Michael Okocha, Carlotta La Raja, Stella Nikolaou, Hannah Cuthbert, Ahmad Nasasra, Carolynne J. Vaizey, Gregory P. Thomas

**Affiliations:** ^1^ Sir Alan Park’s Physiology Unit St Mark's the National Bowel Hospital London UK; ^2^ Institute of Continuing Education Cambridge University Cambridge UK; ^3^ Imperial College London London UK

**Keywords:** faecal incontinence, rechargeable sacral neuromodulation, sacral nerve stimulation, sacral neuromodulation

## Abstract

**Introduction:**

Faecal incontinence (FI) remains a debilitating condition with considerable psychosocial burden. Rechargeable sacral neuromodulation (rSNM) systems offer extended battery longevity and MRI compatibility, potentially reducing surgical burden. However, real‐world data on their efficacy, safety and acceptability remain limited.

**Method:**

This single‐centre observational cohort study evaluated 37 patients who received rSNM (InterStim™ Micro) for FI between August 2020 and August 2022. Patients completed validated questionnaires (St Mark's FI Score, Rockwood FIQOL, SF‐36) and a structured telephone interview assessing functional outcomes, adverse events and device satisfaction. Clinical records were reviewed for surgical and 3‐ to 5‐year follow‐up data.

**Results:**

Median age was 63 years; 61% had a history of obstetric injury. Twenty‐eight patients received rSNM as a replacement for non‐rechargeable systems. Device satisfaction was low: 62% expressed regret and 96% reported challenges with charging. Connectivity issues affected 86% of patients. Six patients underwent device explantation and two required lead revision. Rockwood FIQOL and SF‐36 scores indicated persistent impairment in lifestyle, emotional well‐being and social functioning. Improvements in continence scores were modest and below expectations.

**Conclusion:**

Despite theoretical advantages, rSNM provided limited clinical benefit in this cohort. High rates of dissatisfaction, management difficulties and surgical revisions raise concerns about its broader applicability. While the smaller device may suit select patients, non‐rechargeable systems with extended battery life may represent a more acceptable first‐line option. Further studies are needed to better define the role of rSNM in FI management.

**What does this paper add to the literature?**

This study provides the largest and longest follow‐up evaluation of rechargeable sacral neuromodulation for faecal incontinence, demonstrating modest functional benefit, high device management burden and substantial dissatisfaction. These real‐world data challenge assumptions about rechargeable systems and inform more selective, patient‐centred device choice.

## INTRODUCTION

Faecal incontinence (FI) is a chronic disorder of defaecation, defined by the Rome IV criteria as recurrent involuntary loss of stool for ≥3 months [1]. It is a globally prevalent condition, affecting ~8% of adults and accounts for ~2% of UK healthcare expenditure [2, 3, 4]. Sacral neuromodulation (SNM) is the recommended surgical therapy for adults with FI refractory to conservative treatment, as outlined in contemporary European guidance (UEG/ESCP/ESNM/ESPCG) [5]. Standard practice uses a staged pathway—temporary lead placement with subsequent implantation of a permanent pulse generator (IPG) when ≥50% clinical improvement is achieved [6, 7]. Conventional non‐rechargeable devices are effective [8, 9, 10] but have finite battery life (~4.4 years), mandating replacement operations and incurring cumulative procedural risk and cost [11].

Rechargeable SNM (rSNM) systems were developed to address these limitations. They offer substantially extended battery longevity (up to ~15 years) and full MRI conditionality [12, 13, 14], addressing key limitations of non‐rechargeable systems. These attributes should, in principle, reduce the long‐term procedural burden. However, robust real‐world data on efficacy, device tolerability, maintenance requirements and patient satisfaction remain sparse [15, 16].

### Aim

To evaluate, in a consecutive single‐centre cohort, the efficacy, adverse events and patient‐reported acceptability of rSNM (InterStim™ Micro) for FI, including exploratory subgroup analysis of primary implants versus replacements.

## METHOD

This was a single‐centre observational cohort study conducted at St Mark's National Bowel Hospital, London, UK. Ethical approval was granted via the Health Research Authority Research Ethics Committee (IRAS ID 32715). Consecutive adults who underwent implantation of a rechargeable sacral neuromodulation system (InterStim™ Micro SureScan™, Medtronic, Minneapolis, MN) for faecal incontinence (FI) between August 2020 and August 2022 were eligible. Patients were included if they had an rSNM device and were able to provide verbal consent. Patients with a standard non‐rechargeable system or who declined consent were excluded.

During the study period, when a non‐rechargeable IPG reached end‐of‐life, patients were routinely offered a choice between replacement with a like‐for‐like non‐rechargeable generator (battery life at that time ~ 7–8 years) or conversion to the newer rechargeable system with substantially longer projected battery life and theoretical reduction in future generator exchange procedures. This approach explains the high proportion of replacement implants in this cohort.

A small number of non‐rechargeable InterStim II systems (*n* = 7) were also implanted during the study window. Two of these occurred early in 2020, when there was a brief delay in procurement of the rechargeable platform. Thereafter, patients were counselled on both options, and most elected to proceed with the rechargeable device given its projected generator longevity (~15 years versus ~5–7 years for InterStim II).

### Outcomes

The primary objective was to evaluate efficacy (continence outcomes) and adverse events (e.g. surgical site infection, lead‐related issues, pain and stimulation intolerance). Secondary objectives were patient‐reported satisfaction and device acceptability.

### Procedure

Insertion followed the steps similar to the standard sacral neuromodulation technique described previously [17]. Rechargeable systems use a smaller implantable pulse generator and alternative delivery kits, introducing minor variation in lead handling and generator pocket placement [18]. The intraoperative intent was to obtain stimulation through all available electrodes and programme the lowest effective amplitude. During this period, test phases were performed using percutaneous nerve evaluation.

### Data collection and instruments

Eligible patients were contacted by telephone by a trained investigator. Patients expressing interest were sent a Participant Information Sheet; verbal consent was obtained ≥10 days later and documented in the research file. Telephone interviews were selected to maximize adherence in a population known to have poor return rates for long paper/online questionnaires; interviews followed a fixed structure, and breaks were offered to mitigate burden.

Four questionnaires were administered:

1. A non‐validated study‐specific questionnaire
2. St Mark's Faecal Incontinence Score [19]
3. Rockwood Faecal Incontinence Quality of Life Score (FIQOL [20])
4. The 36‐Item Short Form Health Survey (SF‐36 [21])

In addition to the validated instruments, we developed a brief study‐specific questionnaire to capture domains not measured by standard scores but relevant to rSNM (device handling, charging burden, connectivity problems, adverse stimulation sensations, procedural regret and global device satisfaction). Items were generated by the clinical team based on recurring clinic themes and were piloted in five patients to ensure clarity. This instrument is non‐validated and was used solely for exploratory descriptive purposes. Full item wording and response anchors are provided in Appendix S1.

Clinical data (demographics, FI characteristics and adverse events) were obtained from medical records. Outcomes and analysis approach were prespecified. Continuous variables were presented as median (range) or mean ± standard deviation, depending on distribution (Shapiro–Wilk). Categorical variables were expressed as *n* = or *n* (%). Exploratory subgroup analysis compared primary implants versus replacement implants. All analyses were performed using R (RStudio Team, 2020).

## RESULTS

### Cohort and follow‐up

Between August 2020 and August 2022, 38 patients underwent rSNM implantation, including three males. Thirty‐seven (97.4%) completed ≥24‐month follow‐up interviews; one declined.

### Baseline characteristics

Median age was 63 years (range 37–86) and median BMI was 26.2 kg/m^2^. Obstetric injury was the primary aetiology (61%). Iatrogenic injury (e.g. haemorrhoidectomy or multiple perianal procedures) accounted for 8%. Other causes (10%) included spinal pathology (e.g. cauda equina), congenital anorectal malformation or sequelae of complex pelvic surgery.

Twenty‐eight of 38 (74%) underwent rSNM as a replacement for a prior non‐rechargeable system; 10 (26%) were primary implants. Replacement was undertaken for: battery depletion (*n* = 20), lead displacement (*n* = 4), non‐functioning leads despite correct position (*n* = 2), pain (*n* = 1) and MRI incompatibility (*n* = 1).

Pre‐operative St Mark's FI score (excluding the MRI‐driven replacement) was a median of 18 (range 10–24). Median FI duration was 15 years. Mixed FI was the most common (26/37; 71%), followed by urge (9/37; 24%) and passive (2/37; 5%). Median resting anal pressure was 44 mmHg (range 15–68 mmHg). EAUS was normal in nine patients; the remainder had sphincter defects, scarring or degenerative change.

### Efficacy and adverse events

Median post‐implant St Mark's FI score was 16 (range 7–21). Median deferral time was 5 min (range 2–7).

Eight device‐related reinterventions occurred: six explants and two lead revisions. Reasons for explant included pocket migration (*n* = 1), lead extrusion (*n* = 1) and poor function (*n* = 4).

### Device management and acceptability

Device‐related pain was reported by six patients. Median interval between recharges was 7 days (range 4–10) with median charging duration of 1 h. Median amplitude in use was 2 mA (range 2–3 mA).

Connectivity problems were reported by 75% and broader device management difficulty by 96%. Reported problems included poor charger alignment and frequent disconnections, prolonging recharge sessions.

Patient‐reported scales were standardized to a direction of ‘higher = worse’ prior to analysis. Mean dissatisfaction score was 2.4 (1–5 scale) and 25/37 (68%) rated dissatisfaction at 4–5. Device regret (score 4–5) was present in 20/37 (54%).

### Quality of life assessments

FIQL and SF‐36 data were collected contemporaneously at follow‐up only. FIQL domain means were: Lifestyle 2.88 (SD 0.78); Coping/Behaviour 1.92 (0.61); Depression/Self‐Perception 2.71 (0.74); Embarrassment 1.87 (0.50).

SF‐36 subscale scores demonstrated impairments across physical and psychosocial domains (Table 1).

**TABLE 1:** SF‐36 quality of life mean scores.

SF‐36 domain	Mean	SD
Physical Functioning	71.0	27.6
Role Physical	52.9	44.8
Role Emotional	71.8	38.5
Vitality (Energy/Fatigue)	50.7	21.4
Emotional Well‐being	64.0	24.2
Social Functioning	52.7	21.5
Bodily Pain	57.4	24.6
General Health Perception	53.4	23.9
Health Change	48.5	18.7

### Adverse events

Eight device‐related reinterventions occurred: six full explants and two lead revisions. One patient developed early lead extrusion at 4 weeks, and one experienced generator migration from the pocket. The remaining four explants were undertaken due to poor function. Of the two lead revisions, both were for lead‐related dysfunction without infection. No deep infection, neurological injury or serious stimulation‐related complication was recorded.

## DISCUSSION

This represents the largest reported series of rechargeable sacral neuromodulation (rSNM) for faecal incontinence (FI) to date, with the longest follow‐up duration. In this consecutive cohort, rSNM resulted in only modest improvement in St Mark's continence scores and no clinically meaningful change in deferral time at ≥24 months. Patient‐reported burden was substantial, with very high rates of connectivity problems and charging difficulty, and more than half reporting device regret. These observations contrast with the favourable continence outcomes historically reported with SNM overall [22, 23].

A key contextual factor is case mix: 74% of our cohort were replacement implants. Replacement surgery may yield lower clinical benefit than primary implantation, and our exploratory subgroup comparisons were directionally consistent with this known effect. The limited gains observed here therefore likely reflect both device‐specific characteristics and the underlying population.

Rechargeable systems offer theoretical advantages (up to ~15‐year generator longevity) but were associated with high maintenance demand in practice. Manufacturer guidance suggests charging intervals ranging from ~19 days at 1 mA to ~3 days at 7 mA [12, 13]. In our cohort, patients typically charged weekly for ~1 h, and some required prolonged sessions (90–180 min). Charging issues have been documented previously [24, 25] and were common here. Connectivity and handling problems were near‐universal, with 86% reporting difficulty maintaining connectivity and 92% reporting broader management challenges.

Device‐related adverse events (including pain and system challenges) also contributed to the burden [26, 27]. Collectively, these factors likely drove dissatisfaction and regret, rather than stimulation efficacy alone.

Quality of life instruments (Rockwood FIQOL and SF‐36) demonstrated substantial psychosocial and functional impairment at follow‐up. As pre‐operative QoL was not collected, within‐patient change cannot be inferred; the low scores should be interpreted as a snapshot of the burden in this population.

Since this cohort was implanted, long‐life non‐rechargeable systems (InterStim X™) have become available, with projected longevity approaching rechargeable devices. This development is likely to reduce the rationale for rSNM in routine practice.

On current evidence, rSNM should be reserved for selected indications—for example, individuals who explicitly request a rechargeable generator or those with very low BMI where a smaller IPG profile may confer a technical advantage. For most patients, a long‐life non‐rechargeable system is likely to offer a more acceptable and durable solution.

Future studies should adopt the emerging FI core outcome set, stratify primary vs. replacement implants at the design stage, and incorporate patient workload (charging burden) as a core outcome domain.

## CONCLUSION

In summary, the real‐world performance of rSNM in this cohort was characterized by modest continence benefit and significant device management burden. Given the availability of long‐life non‐rechargeable generators, rSNM should not be considered default therapy. Its use should be restricted to narrow indications and only in fully counselled patients who actively choose a rechargeable platform.

## AUTHOR CONTRIBUTIONS

**C. La Raja:** Methodology; data curation; formal analysis; writing – original draft; investigation. **C. J. Vaizey:** Supervision; writing – review and editing. **G. P. Thomas:** Supervision; conceptualization; methodology; data curation; writing – review and editing; project administration. **H. Cuthbert:** Data curation. **A. Nasasra:** Data curation. **M. Okocha:** Methodology; data curation; formal analysis; writing – original draft. **S. Nikolaou:** Data curation; investigation.

## FUNDING INFORMATION

There are no funders to report for this submission.

## CONFLICT OF INTEREST STATEMENT

The authors declare no competing interests.

## ETHICS STATEMENT

Health Research Authority approval was granted for this study, IRAS ID 327159. Confirmation of capacity and capability was successfully attained at London Northwest University Healthcare Trust prior to study commencement.

## Supporting information


**Appendix S1.** Patient name.

## Data Availability

The data that support the findings of this study are available from the corresponding author upon reasonable request.

## References

[1] Drossman DA , Hasler WL . Rome IV—functional GI disorders: disorders of gut‐brain interaction. Gastroenterology. 2016;150(6):1257–1261. 27147121 10.1053/j.gastro.2016.03.035

[2] Mack I , Hahn H , Gödel C , Enck P , Bharucha AE . Global prevalence of fecal incontinence in community‐dwelling adults: a systematic review and meta‐analysis. Clin Gastroenterol Hepatol. 2024;22(4):712–731.e8. 37734583 10.1016/j.cgh.2023.09.004 PMC10948379

[3] National Institute of Health and Care Excellence . 2018 Surveillance of Faecal Incontinence (NICE Guideline CG49) [Internet]. London: National Institute for Health and Care Excellence (NICE); 2018. Available from: https://www.nice.org.uk/guidance/cg49/resources/2018‐surveillance‐of‐faecal‐incontinence‐nice‐guideline‐cg49‐pdf‐6347633295301 31869032

[4] Ng KS , Sivakumaran Y , Nassar N , Gladman MA . Fecal incontinence: community prevalence and associated factors—a systematic review. Dis Colon Rectum. 2015;58(12):1194–1209. 26544818 10.1097/DCR.0000000000000514

[5] Assmann SL , Keszthelyi D , Kleijnen J , Anastasiou F , Bradshaw E , Brannigan AE , et al. Guideline for the diagnosis and treatment of Faecal incontinence—a UEG/ESCP/ESNM/ESPCG collaboration. UEG J. 2022;10(3):251–286. 10.1002/ueg2.12213 PMC9004250 35303758

[6] Matzel KE , Bittorf B . Sacral neuromodulation for faecal incontinence. Continence. 2024;12:101695.

[7] Saldana Ruiz N , Kaiser AM . Fecal incontinence – challenges and solutions. WJG. 2017;23(1):11–24. 28104977 10.3748/wjg.v23.i1.11 PMC5221273

[8] Axonics . *Axonics R15 Neurostimulator Implant Manual* [Internet]. 2023. Available from: https://www.axonics.com/hcp/resources/resource‐library/

[9] Altomare DF , Giuratrabocchetta S , Knowles CH , Muñoz Duyos A , Robert‐Yap J , Matzel KE , et al. Long‐term outcomes of sacral nerve stimulation for faecal incontinence. Br J Surg. 2015;102(4):407–415. 25644687 10.1002/bjs.9740

[10] Noblett KL , Dmochowski RR , Vasavada SP , Garner AM , Liu S , Pietzsch JB . Cost profiles and budget impact of rechargeable versus non‐rechargeable sacral neuromodulation devices in the treatment of overactive bladder syndrome. Neurourol Urodyn. 2017;36(3):727–733. 27062384 10.1002/nau.23008

[11] Duchalais E , Meurette G , Perrot B , Wyart V , Kubis C , Lehur PA . Exhausted implanted pulse generator in sacral nerve stimulation for faecal incontinence: what next in daily practice for patients? Int J Color Dis. 2016;31(2):439–444. 10.1007/s00384-015-2433-1 26552785

[12] Medtronic . *Therapy Guide* [Internet]. 2020. Report No. UC202002282b EN. Available from: https://asiapac.medtronic.com/content/dam/medtronic‐com/us‐en/patients/treatments‐therapies/bladder/documents/interstim‐micro‐therapy‐guide.pdf

[13] Medtronic . System Eligibility, Battery Longevity, Specifications . 2020. Report No. M988757A003 Rev A.

[14] De Wachter S , Knowles CH , Elterman DS , Kennelly MJ , Lehur PA , Matzel KE , et al. New technologies and applications in sacral neuromodulation: an update. Adv Ther. 2020;37(2):637–643. 31875299 10.1007/s12325-019-01205-z PMC7004424

[15] Jottard K , den Van Broeck S , Komen N , Bruyninx L , De Wachter S . Treatment of fecal incontinence with a rechargeable sacral neuromodulation system: efficacy, clinical outcome, and ease of use—six‐month follow‐up. Neuromodulation Technol Neural Interface. 2021;24(7):1284–1288. 10.1111/ner.13298 33107663

[16] Cohn JA , Kowalik CG , Kaufman MR , Reynolds WS , Milam DF , Dmochowski RR . Evaluation of the axonics modulation technologies sacral neuromodulation system for the treatment of urinary and fecal dysfunction. Expert Rev Med Devices. 2017;14(1):3–14. 27915486 10.1080/17434440.2017.1268913

[17] Dudding TC , Hollingshead JR , Nicholls RJ , Vaizey CJ . Sacral nerve stimulation for faecal incontinence: patient selection, service provision and operative technique: sacral nerve stimulation for faecal incontinence. Color Dis. 2011;13(8):e187–e195. 10.1111/j.1463-1318.2011.02650.x 21689330

[18] Elterman DS . The novel Axonics® rechargeable sacral neuromodulation system: procedural and technical impressions from an initial North American experience [Internet]. Neurourol Urodyn. 2018;37(S2). 10.1002/nau.23482 29336078

[19] Maeda Y , Vaizey CJ , Norton C . St. Mark's incontinence score. Dis Colon Rectum. 2007;50(12):2252. 17899272 10.1007/s10350-007-9076-4

[20] Rockwood TH , Church JM , Fleshman JW , Kane RL , Mavrantonis C , Thorson AG , et al. Fecal incontinence quality of life scale: quality of life instrument for patients with fecal incontinence. Dis Colon Rectum. 2000;43(1):9–16. 10813117 10.1007/BF02237236

[21] Stewart AL , Hays RD , Ware JE . The MOS short‐form general health survey: reliability and validity in a patient population. Med Care. 1988;26(7):724–735. 3393032 10.1097/00005650-198807000-00007

[22] Eggers E , Crouss T , Beausang J , Smith D , Spector S , Saracco B , et al. Long‐term outcomes of sacral nerve stimulation on the treatment of fecal incontinence: a systematic review. Neuromodulation. 2025;28(5):715–726. 10.1016/j.neurom.2024.06.504 39152989

[23] Simillis C , Lal N , Qiu S , Kontovounisios C , Rasheed S , Tan E , et al. Sacral nerve stimulation versus percutaneous tibial nerve stimulation for faecal incontinence: a systematic review and meta‐analysis. Int J Color Dis. 2018;33(5):645–648. 10.1007/s00384-018-2976-z 29470730

[24] McCrery R , Lane F , Benson K , Taylor C , Padron O , Blok B , et al. Treatment of urinary urgency incontinence using a rechargeable SNM system: 6‐month results of the ARTISAN‐SNM study. J Urol. 2020;203(1):185–192. 31347955 10.1097/JU.0000000000000458

[25] Gamé X , Ruffion A , Cornu JN , Phé V , Peyronnet B , Perrouin‐Verbe MA , et al. Sacral neuromodulation: rechargeable versus non‐rechargeable device. What would the patient preferences be in France? Prog Urol. 2022;32(10):672–680. 35752523 10.1016/j.purol.2022.04.011

[26] Pezzella A , McCrery R , Lane F , Benson K , Taylor C , Padron O , et al. Two‐year outcomes of the ARTISAN‐SNM study for the treatment of urinary urgency incontinence using the Axonics rechargeable sacral neuromodulation system. Neurourol Urodyn. 2021;40(2):714–721. 33508155 10.1002/nau.24615 PMC7986436

[27] Blok B , Van Kerrebroeck P , De Wachter S , Ruffion A , Van Der Aa F , Jairam R , et al. Three month clinical results with a rechargeable sacral neuromodulation system for the treatment of overactive bladder [Internet]. Neurourol Urodyn. 2018;37:S9–S16. 10.1002/nau.23465 29315785

