# Supplementary material for: Outcomes of rechargeable sacral neuromodulation for faecal incontinence: A single‐centre observational study
Source: Colorectal Dis. 2025 Dec 21;27(12):e70344. doi: 10.1111/codi.70344 (PMC12719927; doi:10.1111/codi.70344)
Supplement: Supplementary file 1 — Appendix S1. Patient name. [file CODI-27-0-s001.docx]

# APPENDIX 1 Patient name:

Clinical data collected from patients’ records

| **Variable Name** | **Variable details** | **Unit of Measure** |
| --- | --- | --- |
| Age | Age at the time of treatment | years |
| Sex | Sex of patient | - |
| BMI | Body Mass Index | kg/m^2^ |
| Aetiology of FI | FI cause (e.g. obstetric injury, iatrogenic, other) | - |
| History of FI | Time since the onset of FI before SNS implant | years |
| Associated UI | Presence of urinary incontinence symptoms before SNS implant | - |
| Type of FI | i.e. passive/urge/mixed FI | - |
| Pre-implant St Mark’s FI score | St Mark’s FI score before SNS implant | - |
| Pre-implant no. of FI per week | Number of FI episodes before SNS implant | - |
| Pre-implant deferral time | Ability to defer defecation before SNS implant | minutes |
| Pre-implant ARP results | Results of anorectal manometry before SNS implant |  |
|  | Maximum resting pressure | cmH2O |
|  | Peak squeeze increment | cmH2O |
|  | Five second squeeze increment | cmH2O |
|  | Involuntary squeeze incrment | cmH2O |
|  | Anal canal lenght | cm |
|  | Threshold volume of air | ml |
|  | Urge volume of air | ml |
|  | Maximal volume of air | ml |
|  | Mid anal canal electrical sensitivity | mA |
|  | Rectal sensitivity | mA |
|  | Baloon expulsion 50 ml of water | normal/abnormal |
| Pre-implant EAUS | Results of EASU before SNS implant |  |
| Pre-implant BFB | Number of BFB sessions before SNS implant | - |
| PTNS | PTNS trial before SNS implant | - |
| Date of surgery | Date of implantation of rechargeable SNS | dd/mm/yyyy |
| Stimulation amplitude to achieve a motor response | Stimulation amplitude to achieve a motor response at time of surgery | Volts |
| Electrode location | Location of the electrode at the moment of the implant (e.g S3/S4; rirgh/left) | - |
| SSI | SSI after surgery | - |
| Electrode displacement | Displacement of electrode after surgery | - |
| Need for implant removal |  | - |
| Technical failure requiring removal | Malfunctioning or lack of functioning of the SNS implant requiring removal | - |
| Other adverse events requiring removal | Any cause of implant removal | - |
| Need for device revision | Need for further revision surgery | - |
| Number of revisions | Number of revision operations | - |
| Need for follow up outside the planned schedule | Number of follow-up appointments outside the planned schedule | - |
| Date of the last follow-up | Date of the telephone questionnaire | dd/mm/yyyy |

**Abbreviations:** FI faecal incontinence; SNS sacral nerve stimulator; UI urinary incontinence; ARP anorectal physiology; EAUS endoanal ultrasound, PTNS percutaneous tibial nerve stimulation,SSi surgical site infection

**Patient telephone questionnaire**

**Part 1 Non-validated questionnaire**

| **1** | Is the device still in place? | **Yes** | | | **No** | | |
| --- | --- | --- | --- | --- | --- | --- | --- |
| **2** | If device was removed, what was the cause for device removal |  | | | | | |
| **3** | Do you experience pain or discomfort at the implant site? | **Yes** | | | **No** | | |
| **4** | Have you ever experienced adverse stimulation? *(common adverse events related to electrical stimulation include pain, discomfort, transient electric shocks, unwanted vaginal or penile sensation, and unintentional changes in voiding or defecation)* | **Yes** | | | **No** | | |
| **5** | If you experienced adverse stimulation, please specify type, site and number of episodes |  | | | | | |
| **6** | With what frequency do you recharge the device? |  | | | | | |
| **7** | How many minutes does it take for the device to recharge ? |  | | | | | |
| **8** | Do you have any problem in managing the device? | **Yes** | | | **No** | | |
| **9** | If you experience problems in managing the device, please explain |  | | | | | |
| **10** | Do you find it easy to connect the device to the recharger? | **Yes** | | | **No** | | |
| **11** | What therapy settings are you currently using?  *(i.e.amplitude, pulse width, rate of pulses, cycling, intervals on and off therapy)* |  | | | | | |
| **12** | On a scale from 1 to 5, 1 being complete dissatisfaction and 5 complete satisfaction: | | | | | | |
| **12.1** | what is your overall satisfaction with the device? | 1 | 2 | 3 | | 4 | 5 |
| **12.2** | what is your satisfaction with bowel habit/control? | 1 | 2 | 3 | | 4 | 5 |
| **12.3** | do you regret having the device on a scale of 1 to 5? | 1 | 2 | 3 | | 4 | 5 |
| **13** | Do you regret having undergone the procedure? | **Yes** | | | **No** | | |
| **14** | Did the results of the procedure meet your expectations? |  | | |  | | |
| **15** | For how many minutes are you able to defer defecation? |  | | | | | |
| **16** | Do you have problems with bladder control? |  | | | | | |

**Part 2 St.Mark’s Incontinence Score**


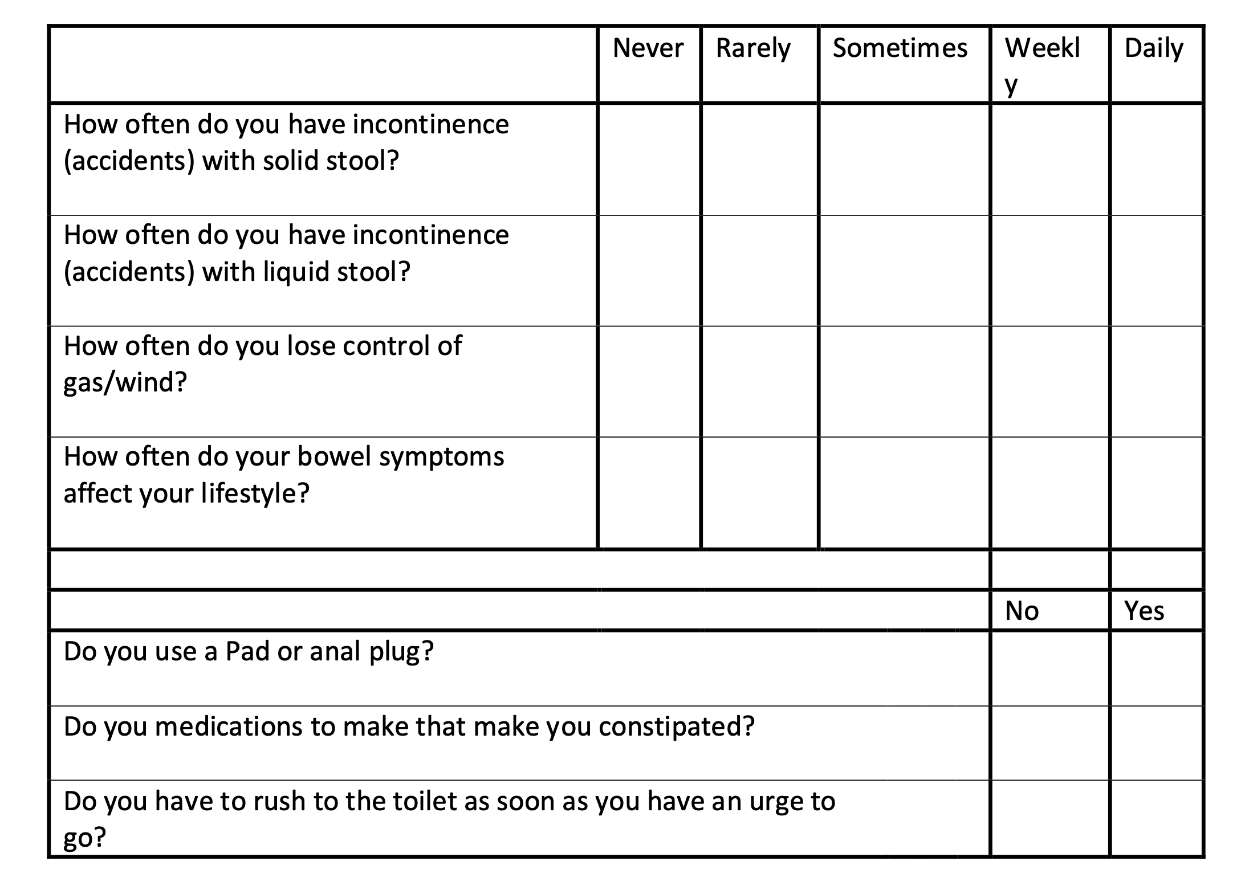


**Part 3 Rockwood Score – Faecal Incontinence Quality of Life Score (FIQOL Score)**

| **1** | In general would you say your health is | **Exellent** | | | Very good | | Good | | | Fair | | | Poor | |
| --- | --- | --- | --- | --- | --- | --- | --- | --- | --- | --- | --- | --- | --- | --- |
| **2**  For each item, how much og the time the issue is a concern for you due to accidental bowel leakage | | | | | | | | | | | | | | |
|  | Due to accidental bowel leakage: | **Most of the time** | | **Some of the time** | | | | **A little of the time** | | | | **None of the time** | | |
| **a** | I am afraid to go out |  | |  | | | |  | | | |  | | |
| **b** | I avoid visiting friends |  | |  | | | |  | | | |  | | |
| **c** | I avoid staying overnight away from home |  | |  | | | |  | | | |  | | |
| **d** | It is difficult for me to get out and do things like going to a movie or to church |  | |  | | | |  | | | |  | | |
| **f** | I cut down how much I eat before I go out |  | |  | | | |  | | | |  | | |
| **g** | Whenever I am away from home I try to stay near a restroom as much as possible |  | |  | | | |  | | | |  | | |
| **h** | I avoid travelling |  | |  | | | |  | | | |  | | |
| **i** | I worry about not being able to get to the toilet in time |  | |  | | | |  | | | |  | | |
| **j** | I feel I have no control over my bowels |  | |  | | | |  | | | |  | | |
| **k** | I can’t hold my bowel movement long enough to get to the bathroom |  | |  | | | |  | | | |  | | |
| **l** | I leak stool without even knowing it |  | |  | | | |  | | | |  | | |
| **m** | I try to prevent bowel accidents by staying very near to a bathroom |  | |  | | | |  | | | |  | | |
| **3** | Due to accidental bowel leakage indicate the extent to which you AGREE or DISAGREE with each of the following items | | | | | | | | | | | | | |
|  | Due to accidental bowel leakage | **Strongly Agree** | | **Somewhat Agree** | | | | **Somewhat Disagree** | | | | **Strongly Disagree** | | |
| **a** | I feel ashamed |  | |  | | | |  | | | |  | | |
| **b** | I cannot do many things I want to do |  | |  | | | |  | | | |  | | |
| **c** | I worry about bowel accidents |  | |  | | | |  | | | |  | | |
| **d** | I feel depressed |  | |  | | | |  | | | |  | | |
| **e** | I worry about others smelling stool on me |  | |  | | | |  | | | |  | | |
| **f** | I feel like I am not a health person |  | |  | | | |  | | | |  | | |
| **g** | I enjoy life less |  | |  | | | |  | | | |  | | |
| **h** | I have sex less often I would like to |  | |  | | | |  | | | |  | | |
| **i** | I feel different from other people |  | |  | | | |  | | | |  | | |
| **j** | The possibility of bowel accidents is always on my mind |  | |  | | | |  | | | |  | | |
| **k** | I am afraid to have sex |  | |  | | | |  | | | |  | | |
| **l** | I avoid travelling by plane or train |  | |  | | | |  | | | |  | | |
| **m** | I avoid going out to eat |  | |  | | | |  | | | |  | | |
| **n** | When I go someplace new, I specifically locate where the bathrooms are |  | |  | | | |  | | | |  | | |
| **4** | During the past month, have you felt so sad, discouraged, hopeless or had so many problems that you wondered if anything was worthwhile? | Extremely so | Very much so | | | Quite a bit | | | Some-enough to bother me | | A little bit | | | Not at all |

**Part 4 36-Item Short Form Survey Instrument**

| **1** | In general, would you say your health is | Excellent | | | | Very good | | | | | | | | | Good | | | | | | | Fair | | | | | | | | Poor |
| --- | --- | --- | --- | --- | --- | --- | --- | --- | --- | --- | --- | --- | --- | --- | --- | --- | --- | --- | --- | --- | --- | --- | --- | --- | --- | --- | --- | --- | --- | --- |
| **2** | Compared to one year ago, how would you rate your health in general now? | Much better | | | | Somewhat better | | | | | | | | | About the same | | | | | | | Somewhat worse | | | | | | | | Much worse |
| The following items are about activities you might do during a typical day. Does your health now limit you in these activities? If so, how much? | | | | | | | | | | | | | | | | | | | | | | | | | | | | | | |
|  |  | **Yes, a lot** | | | | | | | **Yes, a little** | | | | | | | | | | | | | | | **No** | | | | | | |
| **3** | Vigorous activities, such as running, lifting heavy objects, participating in strenuous sports |  | | | | | | |  | | | | | | | | | | | | | | |  | | | | | | |
| **4** | Moderate activities, such as moving a table, pushing a vacuum cleaner, bowling, or playing golf |  | | | | | | |  | | | | | | | | | | | | | | |  | | | | | | |
| **5** | Lifting or carrying groceries |  | | | | | | |  | | | | | | | | | | | | | | |  | | | | | | |
| **6** | Climbing several flights of stairs |  | | | | | | |  | | | | | | | | | | | | | | |  | | | | | | |
| **7** | Climbing one flight of stairs |  | | | | | | |  | | | | | | | | | | | | | | |  | | | | | | |
| **8** | Bending, kneeling, or stooping |  | | | | | | |  | | | | | | | | | | | | | | |  | | | | | | |
| **9** | Walking more than a mile |  | | | | | | |  | | | | | | | | | | | | | | |  | | | | | | |
| **10** | Walking several blocks |  | | | | | | |  | | | | | | | | | | | | | | |  | | | | | | |
| **11** | Walking one block |  | | | | | | |  | | | | | | | | | | | | | | |  | | | | | | |
| **12** | Bathing or dressing yourself |  | | | | | | |  | | | | | | | | | | | | | | |  | | | | | | |
| During the past 4 weeks, have you had any of the following problems with your work or other regular daily activities as a result of your physical health? | | | | | | | | | | | | | | | | | | | | | | | | | | | | | | |
|  |  | **Yes** | | | | | | | | | | | | | | **No** | | | | | | | | | | | | | | |
| **13** | Cut down the amount of time you spent on work or other activities |  | | | | | | | | | | | | | |  | | | | | | | | | | | | | | |
| **14** | Accomplished less than you would like |  | | | | | | | | | | | | | |  | | | | | | | | | | | | | | |
| **15** | Were limited in the kind of work or other activities |  | | | | | | | | | | | | | |  | | | | | | | | | | | | | | |
| **16** | Had difficulty performing the work or other activities (for example, it took extra effort) |  | | | | | | | | | | | | | |  | | | | | | | | | | | | | | |
| During the past 4 weeks, have you had any of the following problems with your work or other regular daily activities as a result of any emotional problems (such as feeling depressed or anxious)? | | | | | | | | | | | | | | | | | | | | | | | | | | | | | | |
| **17** | Cut down the amount of time you spent on work or other activities |  | | | | | | | | | | | | | |  | | | | | | | | | | | | | | |
| **18** | Accomplished less than you would like |  | | | | | | | | | | | | | |  | | | | | | | | | | | | | | |
| **19** | Didn't do work or other activities as carefully as usual |  | | | | | | | | | | | | | |  | | | | | | | | | | | | | | |
| **20** | During the past 4 weeks, to what extent has your physical health or emotional problems interfered with your normal social activities with family, friends, neighbors, or groups? | not at all | | | slightly | | | | | | | moderately | | | | | | | | | quite a bit | | | | | extremely | | | | |
| **21** | How much bodily pain have you had during the past 4 weeks? | none | very mild | | | | | | | mild | | | | | | | moderate | | | | | | | | severe | | | | very severe | |
| **22** | During the past 4 weeks, how much did pain interfere with your normal work (including both work outside the home and housework)? | not at all | | | | | a little bot | | | | | | moderately | | | | | | | quite a bit | | | | | | | extremely | | | |
| These questions are about how you feel and how things have been with you during the past 4 weeks. For each question, please give the one answer that comes closest to the way you have been feeling | | | | | | | | | | | | | | | | | | | | | | | | | | | | | | |
|  |  | **All of the time** | | **Most of the tome** | | | | | | | **A good bit of the time** | | | | | | | **Some of the time** | | | | | **A little of the time** | | | | | **None of the time** | | |
| **23** | Did you feel full of pep? |  | |  | | | | | | |  | | | | | | |  | | | | |  | | | | |  | | |
| **24** | Have you been a very nervous person? |  | |  | | | | | | |  | | | | | | |  | | | | |  | | | | |  | | |
| **25** | Have you felt so down in the dumps that nothing could cheer you up? |  | |  | | | | | | |  | | | | | | |  | | | | |  | | | | |  | | |
| **26** | Have you felt calm and peaceful |  | |  | | | | | | |  | | | | | | |  | | | | |  | | | | |  | | |
| **27** | Did you have a lot of energy? |  | |  | | | | | | |  | | | | | | |  | | | | |  | | | | |  | | |
| **28** | Have you felt downhearted and blue? |  | |  | | | | | | |  | | | | | | |  | | | | |  | | | | |  | | |
| **29** | Did you feel worn out? |  | |  | | | | | | |  | | | | | | |  | | | | |  | | | | |  | | |
| **30** | Have you been a happy person? |  | |  | | | | | | |  | | | | | | |  | | | | |  | | | | |  | | |
| **31** | Did you feel tired? |  | |  | | | | | | |  | | | | | | |  | | | | |  | | | | |  | | |
| **32** | During the past 4 weeks, how much of the time has your physical health or emotional problems interfered with your social activities (like visiting with friends, relatives, etc.)? | All of the time | | | | | | Most of the time | | | | | | Some of the time | | | | | A little of the time | | | | | | | None of the time | | | | |
| How TRUE or FALSE is each of the following statements for you. | | | | | | | | | | | | | | | | | | | | | | | | | | | | | | |
| **33** | I seem to get sick a little easier than other people | Definitely true | | | | | | Mostly true | | | | | | Don’t know | | | | | Mostly false | | | | | | | Definitely false | | | | |
| **34** | I am as healthy as anybody I know |  | | | | | |  | | | | | |  | | | | |  | | | | | | |  | | | | |
| **35** | I expect my health to get worse |  | | | | | |  | | | | | |  | | | | |  | | | | | | |  | | | | |
| **36** | My health is excellent |  | | | | | |  | | | | | |  | | | | |  | | | | | | |  | | | | |
